# Editorial: Carbon sequestration and climate change in crops, natural vegetation, and wetland dynamics in the high Andes

**DOI:** 10.3389/fpls.2023.1338577

**Published:** 2023-11-29

**Authors:** Bárbara Vento, Francisco Cuesta, Lohengrin Cavieres

**Affiliations:** ^1^ Laboratorio de Geobotánica y Fitogeografía, National Scientific and Technical Research Council - Argentina (CONICET) Mendoza, Mendoza, Argentina; ^2^ Grupo de Investigación en Biodiversidad, Medio Ambiente y Salud, Universidad de Las Américas, Quito, Ecuador; ^3^ Laboratorio de Invasiones Biólogicas, University of Concepcion, Concepcion, Chile

**Keywords:** Andes, climate change, conservation, biodiversity, wetlands

The Andes, one of the world’s most extensive mountain ranges, stretches across a vast latitudinal range from tropical to subpolar regions along the western side of South America (Clapperton, 1993). This extensive distribution gives rise to diverse ecosystems, including natural forests, shrublands, grasslands, and wetlands. Notably, at elevations beyond the upper limit of tree growth (i.e., treeline), high-altitude wetlands emerge as the most productive ecosystems in the Andes. They provide relevant natural contributions as freshwater for human settlements or livestock and constitute habitats for particular biodiversity in contrast with other regional Andean landscapes. In addition, high-Andean wetlands are important in climate and biogeochemical cycles regulation (Mitsch and Gosselink, 2015). Climate change is already impacting ecosystems worldwide, and it is expected to continue or accelerate its impact in the future, representing a major threat to high mountain ecosystems worldwide (Pauli and Halloy, 2019; Cuesta et al., 2023). Andean wetland ecosystem dynamics are mainly regulated by temperature and precipitation, a factor that places them among the most sensitive and vulnerable ecosystems facing global climate change (Dangles et al., 2017; Cuesta et al., 2019). Moreover, they store considerable amounts of carbon, acting as important global sinks (Hribljan et al., 2016; Alavi-Murillo et al., 2022). Carbon storage in soil and plants growing in these environments play a fundamental role to mitigate global warming, reducing emissions of CO_2_ into the atmosphere (Mitsch et al., 2013; Hribljan et al., 2017).

This Research Topic compiles some of the latest insights into water resources, environmental factors, and climate variables, along with their influence in biological vegetation dynamics within Andean ecosystems. The published manuscripts advance our understanding of ecological patterns and processes across these diverse environments, with a particular emphasis on complex interplay between local plant communities and their surrounding environment. The contributions encompass a broad spectrum of topics, including novel remote mapping techniques, remote sensing applications, carbon cycle dynamics, water balance assessments, natural crop studies, assessment of vegetation diversity trends, and in-depth climate change and its effects on vegetation. This Research Topic provides a comprehensive and up-to-date overview of the complex interactions shaping Andean ecosystems.

The contribution of wetlands in the Huascarán National Park, Perú to carbon stored in soils has been estimated by Chimner et al. in more than 20 Tg of carbon stored in peatlands, representing 97% of the total carbon stored on wetlands. As estimations of carbon stocks in restricted-access areas is difficult, the authors proposed and tested a peat sampling protocol that facilitates field sampling in remote areas. Importantly, the structure and growth of plants are significant factors in the functioning of peatlands as a source of organic matter and protective soil layer.

New insights into the structure and functioning of high-elevation peatlands of the páramos of the northern Andes in Ecuador are shown by Suarez et al. Three distinct petland vegetation types were identified, and elevation and co-occurrent environmental changes such as temperature and soil age played a significant role in the structure, aboveground biomass pattern, and composition of these peatlands.

The study conducted by Castillo-Figueroa et al. deals with wood density as a relevant functional trait related to ecological strategies and ecosystem carbon dynamics in the tropical Andean forests of Colombia. They quantified the wood density of different tree and shrub species to determine how wood density modifies due to forest succession and its relationship to primary productivity. Their findings reveal that wood density is strongly related to the succession process and carbon stored as biomass and productivity. The insights provided by this study are important for comprehending Andean forests’ recovery pathways following a detrimental event and hold conservation significance for informing restoration practices.

Nitrogen is a macronutrient directly involved in plant’s carbon assimilation. The study of Jerez et al. evaluated the effects of different nitrogen sources on the performance of *Chenopodium quinoa*, a native Andean crop species. They measured a set of ecophysiological variables in plants from the north and south part of the Chilean Andes growing with different nitrogen sources (nitrate and ammonium). The authors highlight the role of nitrogen source on triggering population-specific responses that are relevant to our understanding of biomass accumulation and the respiration process in Andean species.

The utilization of remote sensing techniques, as demonstrated in the work by Carilla et al., has facilitated an in-depth exploration of vegetation dynamics in response to a shifting climate within the high Andes of northwestern Argentina. This research entailed a comprehensive analysis of Andean vegetation over an extensive temporal scope coupled with an assessment of climate and hydrological variables across varying scales. In doing so, the authors have introduced a practical, accessible, and valuable long-term monitoring tool for observing mountain ecosystems, which integrates biological and physical processes and their synergistic interplay.

Global climate change, accelerated by human activity, is notoriously changing ecosystem functioning, increasing shifts in biotic communities, and leading to biodiversity loss at various spatial scales. Global change stressors could disrupt hydrological, climatic, biological, and ecological processes, increasing the uncertainty in ecosystem services provision. All the contributions collected in this Research Topic reflect the interest in understanding and quantifying the interactions between hydrological, ecological, and biogeochemical processes that take place in wetland ecosystems. The scientific knowledge generated through these investigations is essential information to enhance our understanding of Andean ecosystems and provide a valuable foundation for informing conservation and management practices.

## Author contributions

BV: Writing – original draft, Writing – review & editing. FC: Writing – review & editing. LC: Writing – review & editing.
